# MEETINGS CALENDAR - 2017

**Published:** 2017

## March

### 8 - Current Concepts in Pediatric and Adult Congenital Cardiac Critical Care

New York, United States

Informations: Arthur J. Smerling, MD

E-mail: ajs8@cumc.columbia.edu

Site:register.columbiacme.org/conference.cgi?rm=view&conference_id=814481

### 10 - AATS Grant Writing Workshop

Bethesda, United States

Informations: American Association for Thoracic Surgery

Phone: 978-252-2200

E-mail: meetings@aats.org

Site: www.aats.org/grantwriting

### 12 to 14 - SCTS Annual Meeting & Cardiothoracic Forum 2017

Belfast, United Kingdom

Informations: Isabelle Ferner

Phone: +44 (0)7949211636

E-mail: sctsadmin@scts.org

Site: www.scts.org

### 13 to 18 - ESTS Knowledge Track “Antalya Revisited in Prague”

Prague, Czech Republic

Informations: Sue Hesford

Phone: + 44 7841519795

Fax: + 44 1392 430671

E-mail: sue@ests.org.uk

Site: www.ests.org

### 15 - 2nd International Conference on titleardiac Surgery

London, United Kingdom

Informations: Ileana Peter

Phone: 702-508-5200

E-mail: cardiovascular@cardiologyconference.org

Site: cardiovascular.conferenceseries.com

### 16 to 18 - Introduction to Aortic Surgery

Windsor, United Kingdom

Informations: EACTS

Phone: + 44 7841519795

Fax: + 44 1392 430671

E-mail: info@eacts.co.uk

Site: www.eacts.org/academy/2017-programme/

### 18 to 25 - 35th Cardiovascular Surgical Symposium

Zürs am Arlberg, United Kingdom

Informations: Beatrix Seckl

Phone: +43 664 88 671 571

Fax: +43 274 222 210 015

E-mail: office@conventive.at

Site: www.cardiovascular.at

### 22 to 24 - Master Class on Aortic Valve Repair: A Step-by-Step Approach

Paris, France

Informations: EACTS

E-mail: info@eacts.co.uk

Site: www.eacts.org

### 23 to 26 - The 25th Annual Meeting of the Asian titlend Thoracic Surgery (ASCVTS 2017)

Seoul, South Korea

Informations: Jiwon Yoon

Phone: +82-2-3452-0507

Fax: +82-2-521-8683

E-mail: reg@ascvts2017.org

Site: www.ascvts2017.org

### 23 to 26 - 13th International Congress of Update titleardiovascular Surgery

Izmir, Turkey

Informations: Prof. Dr. Oztekin Oto

Phone: 0090 532 701 4241

Fax: 0090 4642470

E-mail: oztekinoto@oztekinoto.com

Site: www.uccvs2017.org

### 27 to 31 - Thoracic Surgery: Part I Windsor, United Kingdom

Informations: EACTS

E-mail: info@eacts.co.uk

Site: www.eacts.org/academy/2017-programme/

## April

### 31 and 01 - Symposium on Robotic Mitral Valve Repair

Chicago, United States

Informations: Michelle Taylor

Site: www.sts.org/robotics

### 03 and 07 – Houston Methodist CMR Workshop

Houston, United States

Informations: CME

Phone: 713.441.4971

E-mail: cme@houstonmethodist.org

Site: events.houstonmethodist.org/cmri

### 03 – Donor Heart and Lung Procurement Simulation Lab sponsored by UPMC

La Jolla, United States

Informations: Angela Kinnunen

E-mail: www.ctsurgery.pitt.edu/

### 04 and 05 – Cardiomersion 2017

Cairo, Egypt

Informations: Dr. Deepak Puri

Phone: +919814332901

E-mail: drdeephearts@gmail.com

Site: www.cardiomersion.com

### 04 to 06 – 23th Annual Conference of the titleardiothoracic Surgery

Cairo, Egypt

Informations: Sameh M. Elameen

Phone: +201001413339

E-mail: sameh.elameen@gmail.com

Site: www.escts2017.com

### 06 and 07 – Re-Evolution Summit - MICS

Houston, United States

Informations: CME

Phone: 713-441-4971

E-mail: cme@houstonmethodist.org

Site: https://events.houstonmethodist.org/reevolution

### 08 and 09 – Toronto Anesthesia Symposium

Toronto, Canada

Informations: Nancy Bush

E-mail: info-ANS1705@cpdtoronto.ca

Site: www.cpd.utoronto.ca/anesthesia/

### 18 and 19 - 11th World Congress on titleongenital Cardiovascular Disease

London, United Kingdom

Informations: Sharon Williams

Phone: 7025085200

E-mail: sw124143@gmail.com

Site: pediatriccardiology.conferenceseries.com/europe/

### 19 to 21 - 32nd EACTA Annual Congress 2017

Berlin, Germany

Informations: Randi Wilson

E-mail: eacta@ics.dk

Site: www.eacta.org/congress-events/calendar-of-events/

### 19 to 21 - ESTS Skill Track Course “Elancourt in

Copenhagen, Denmark

Informations: Sue Hesford

Phone: + 44 7841519795

Fax: + 44 1392 430671

E-mail: sue@ests.org.uk

Site: www.ests.org/

### 20 and 22 – 44^th^ Congress of the titleardiovascular Surgery

Rio de Janeiro, Brazil

Informations: Brazilian Society of Cardiovascular Surgery

Phone: +55 (11) 3849-0341

E-mail: sbccv@sbccv.org.br

Site: sbccv.org.br/44congresso

### 20 and 21 - 12th Vienna Interdisciplinary titleepair (VISAR)

Vienna, Austria

Informations: Marek Ehrlich

Phone: +4314040056200

E-mail: marek.ehrlich@meduniwien.ac.at

Site: www.visar.at

### 27 and 28 - AATS Mitral Conclave

New York, United States

Informations: The American Association for Thoracic Surgery

Phone: 978-252-2200

Fax: 978-522-8469

E-mail: meetings@aats.org

Site: aats.org/mitral

### 29 - AATS Innovation Summit

Boston, United States

Informations: American Association for Thoracic Surgery

Phone: 978-252-2200

E-mail: meetings@aats.org

Site: www.aats.org/innovation

### 29 to 03 - AATS Centennial

Boston, United States

Informations: American Association for Thoracic Surgery

Phone: 978-252-2200

E-mail: meetings@aats.org

Site: http://www.aats.org/centennial

## May

### 05 - EuroGUCH 2017 Meeting - The Annual Meeting of the ESC Working Group

Lausanne, Switzerland

Informations: Paragon Group

Phone: +41225330948

Fax: +41 (0) 22 5802 953

E-mail: GTito@paragong.com

### 05 to 07 - Southwest Valve Summit 2017

San Antonio, United States

Informations: CME

Phone: 713.441.4971

E-mail: cme@houstonmethodist.org

### 25 and 26 - Massachusetts General Hospital Postgraduate titlehoracic Surgery

Cambridge, United States

Informations: Harvard Medical School

Phone: (617) 384-8600

E-mail: ceprograms@hms.harvard.edu

### 28 to 31 - 25th European Conference on General Thoracic Surgery

Innsbruck, Austria

Informations: Sue Hesford

Phone: + 44 1392 430671

E-mail: sue@ests.org.uk

Site: www.ests.org

